# Effect of tungstate on acetate and ethanol production by the electrosynthetic bacterium *Sporomusa ovata*

**DOI:** 10.1186/s13068-016-0576-0

**Published:** 2016-08-04

**Authors:** Fariza Ammam, Pier-Luc Tremblay, Dawid M. Lizak, Tian Zhang

**Affiliations:** 1 The Novo Nordisk Foundation Center for Biosustainability, Technical University of Denmark, 2970 Hørsholm, Denmark; 2 School of Chemistry, Chemical Engineering and Life Science, Wuhan University of Technology, Wuhan, 430070 People’s Republic of China

**Keywords:** Microbial electrosynthesis, Gas fermentation, Medium optimization, *Sporomusa ovata*, Aldehyde ferredoxin oxidoreductase

## Abstract

**Background:**

Microbial electrosynthesis (MES) and gas fermentation are bioenergy technologies in which a microbial catalyst reduces CO_2_ into organic carbon molecules with electrons from the cathode of a bioelectrochemical system or from gases such as H_2_. The acetogen *Sporomusa ovata* has the capacity of reducing CO_2_ into commodity chemicals by both gas fermentation and MES. Acetate is often the only product generated by *S. ovata* during autotrophic growth.

**Results:**

In this study, trace elements in *S. ovata* growth medium were optimized to improve MES and gas fermentation productivity. Augmenting tungstate concentration resulted in a 2.9-fold increase in ethanol production by *S. ovata* during H_2_:CO_2_-dependent growth. It also promoted electrosynthesis of ethanol in a *S. ovata*-driven MES reactor and increased acetate production 4.4-fold compared to unmodified medium. Furthermore, fatty acids propionate and butyrate were successfully converted to their corresponding alcohols 1-propanol and 1-butanol by *S. ovata* during gas fermentation. Increasing tungstate concentration enhanced conversion efficiency for both propionate and butyrate. Gene expression analysis suggested that tungsten-containing aldehyde ferredoxin oxidoreductases (AORs) and a tungsten-containing formate dehydrogenase (FDH) were involved in the improved biosynthesis of acetate, ethanol, 1-propanol, and 1-butanol. AORs and FDH contribute to the fatty acids re-assimilation pathway and the Wood–Ljungdahl pathway, respectively.

**Conclusions:**

This study presented here shows that optimization of microbial catalyst growth medium can improve productivity and lead to the biosynthesis of different products by gas fermentation and MES. It also provides insights on the metabolism of biofuels production in acetogens and demonstrates that *S. ovata* has an important untapped metabolic potential for the production of other chemicals than acetate via CO_2_-converting bioprocesses including MES.

**Electronic supplementary material:**

The online version of this article (doi:10.1186/s13068-016-0576-0) contains supplementary material, which is available to authorized users.

## Background

Acetogens are anaerobic bacteria capable of growing autotrophically by conducting gas fermentation with H_2_:CO_2_ as well as with synthesis gas (H_2_:CO:CO_2_) [1]. Another promising feature of acetogens is the ability of some to accept electrons from the cathode of a bioelectrochemical system (BES) to reduce CO_2_ into multicarbon compounds in a process named microbial electrosynthesis (MES) [2–4]. Gas fermentation is a promising bioproduction process for recycling industrial gas wastes into commodity chemicals, thus limiting greenhouse gas emissions [5]. MES has additional attractive features. It can be used to store electricity surplus from the power grid into the chemical bonds of valuable products like biofuels [4]. It can also be coupled with solar panels to become an artificial bioinorganic photosynthesis apparatus with a solar-to-chemicals conversion efficiency significantly higher than biomass-based bioproduction technologies [2]. Under autotrophic growth conditions, including gas fermentation and MES, acetogens utilize the acetyl-CoA/Wood–Ljungdahl pathway (WL) to reduce CO_2_ to acetyl-CoA, a precursor in the synthesis of cellular components as well as in the generation of organic carbon products such as acetate and ethanol [1, 6, 7].

One successful approach to improve the production of organic carbon molecules from CO_2_ by acetogens consists of optimizing the cultivation medium composition [8–10]. For example, ethanol production by the acetogen *Clostridium ragsdalei* was improved up to fivefold by optimizing traces elements concentration in the growth medium [11]. This approach also led to increase in bacterial growth and acetate production.

Trace elements are mainly required by bacteria because of their role as cofactors for enzymes involved in metabolic pathways such as the WL pathway. In acetogens, key metalloenzymes of the WL pathway include hydrogenases, the formate dehydrogenase (FDH) and the bifunctional CO dehydrogenase (CODH)/acetyl-CoA synthase (ACS) [12]. Hydrogenases perform the reversible oxidation of molecular H_2_ and require iron only or both iron and nickel [11, 13]. FDHs catalyze the reversible conversion of CO_2_ to formate [14]. They contain tungsten, iron, selenium, and/or molybdenum, depending on the bacterial species and on the availability of these elements in the growth medium [15, 16]. CODH/ACS is a bifunctional enzyme containing nickel and iron catalyzing the reversible reduction of CO_2_ to CO and the synthesis of acetyl-CoA [12, 17, 18].

Acetogens producing or oxidizing the solvent ethanol possess other important metalloenzymes including alcohol dehydrogenases (ADH) catalyzing the reversible oxidation of alcohols and requiring zinc or iron for their activity [19, 20]. Due to energetic considerations, ethanol production by acetogens is thought to occur via acetate re-assimilation [21]. In addition to ADH, this pathway also requires a tungsten-containing aldehyde ferredoxin oxidoreductase (AOR) catalyzing the reduction of acetate to acetaldehyde with ferredoxin as the low-potential electron donor [22–24].

A promising platform for the production of multicarbon compounds from CO_2_ is the Gram-negative bacterium *Sporomusa ovata*, one of the most efficient acetogenic MES microbial catalysts for the production of acetate reported until now [4, 25, 26]. In this study, we attempted to develop the biosynthesis of ethanol by *S. ovata* and to improve acetate production rate by optimizing trace elements in the cultivation medium. Production of acetate and ethanol from CO_2_ with H_2_ or a cathode as the electron source as well as growth under autotrophic condition was investigated with different concentrations of trace elements (WO_4_^2−^, MoO_4_^2−^, SeO_4_^2−^, Ni^2+^, Zn^2+^, and Fe^2+^) in the medium. Furthermore, transcript abundance of genes coding for critical metalloenzymes involved in the WL pathway or in the synthesis of ethanol like AORs was studied to understand better the molecular mechanisms responsible for the improved growth and productivity caused by changes in trace elements concentration. The conversion of longer carbon chain fatty acids than acetate to alcohols during gas fermentation was also investigated to determine if *S. ovata* has the metabolic capacity to synthesize longer alcohols than ethanol.

## Results and discussion

### Impact of varying trace elements concentration on acetate and ethanol production by *S. ovata*

**Fig. 1:**
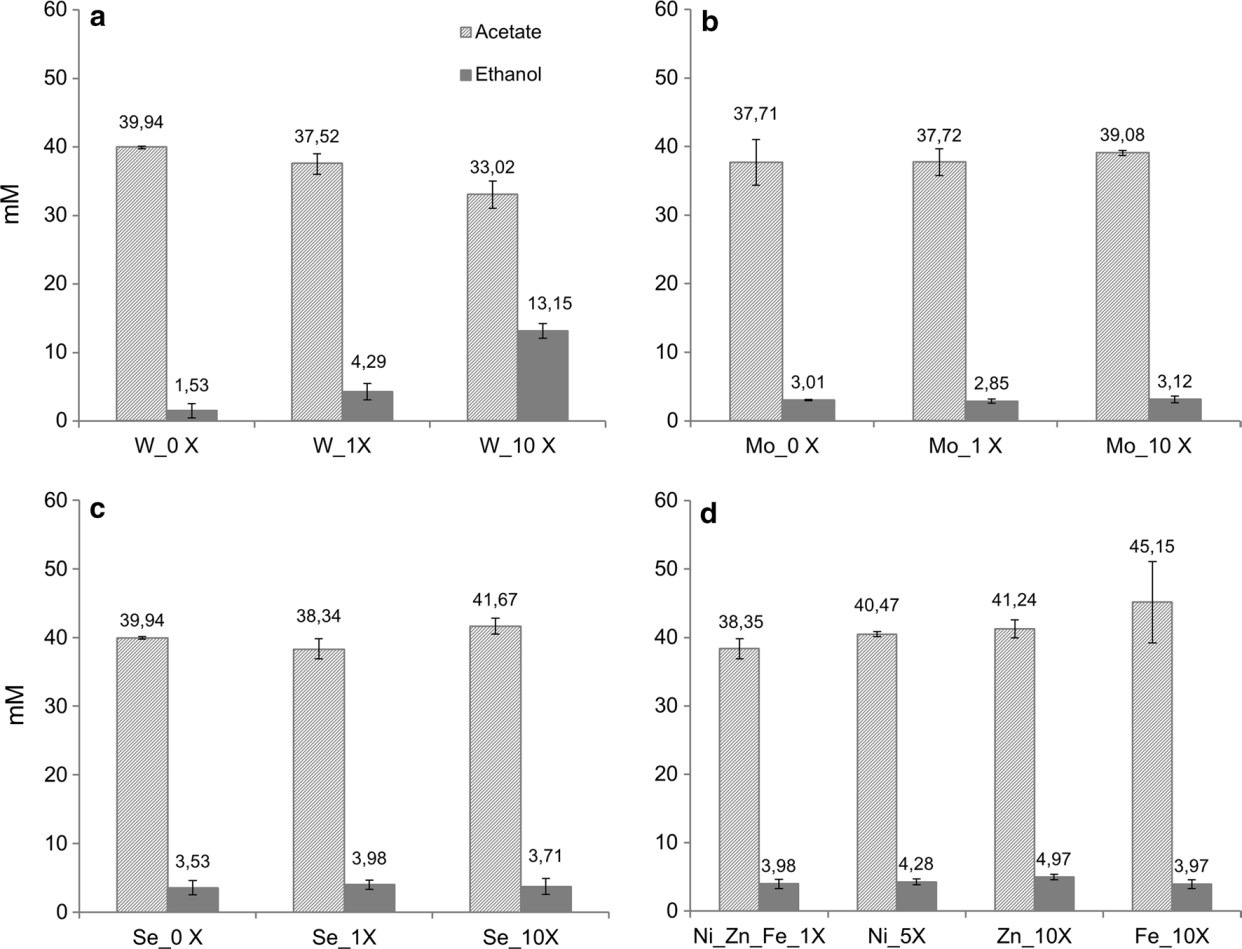
Impact of trace elements on the production of acetate and ethanol by H_2_:CO_2_-grown *S. ovata*. **a** tungstate, **b** molybdate, **c** selenate, and **d** nickel, zinc, and iron. Results shown are from at least three independent experiments

Optimization of the concentration of different trace elements was investigated with *S. ovata* under H_2_:CO_2_ growth condition. Among all the tested elements (WO_4_^2−^, MoO_4_^2−^, SeO_4_^2−^, Ni^2+^, Zn^2+^, and Fe^2+^), only augmentation of tungstate (WO_4_^2−^) concentration had a significant impact on acetate and ethanol production by *S. ovata* after 8 days of growth (Fig. 1). The absence of statistically significant changes in acetate and ethanol production in the presence of augmented concentrations of MoO_4_^2−^, SeO_4_^2−^, Ni^2+^, Zn^2+^, or Fe^2+^ indicates that these elements are already present in sufficient concentrations in the standard 311 medium for *S. ovata*.

Multiplying tungstate concentration by 10 to 0.1 μM in *S. ovata* 311 growth medium resulted in a 3.1-fold increase in ethanol production compared to standard 311 medium (1× tungstate) and a 8.6-fold increase versus 311 medium without added tungstate (0×) (Fig. 1a). These results indicate that optimal ethanol production by *S. ovata* is dependent on tungstate concentration. In contrast, acetate production in the presence of 10× tungstate was reduced by 12.0 ± 9.1 % versus standard medium and 17.3 ± 5.4 % compared to medium with 0× tungstate (Fig. 1a), which indicates that part of the acetate may have been re-assimilated by *S. ovata* to produce ethanol.

### Impact of tungstate on autotrophic growth and production of ethanol and acetate by *S. ovata*

**Fig. 2:**
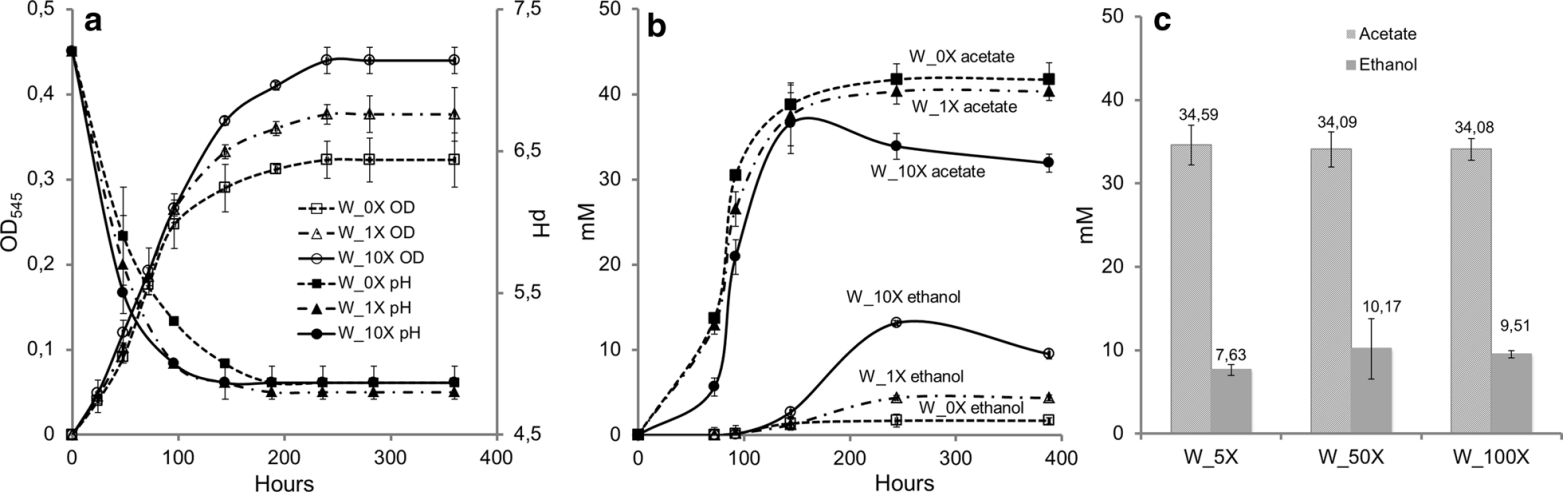
Impact of tungstate concentration on the metabolism of H_2_:CO_2_-grown *S. ovata*. **a** Growth, pH variation and **b** production of acetate and ethanol in the presence of 0×, 1× and 10× tungstate. **c** Production of acetate and ethanol in the presence of 5×, 50× and 100× tungstate. Results shown are from at least three independent experiments

The effect of varying tungstate concentration on growth as well as on ethanol and acetate production was further investigated via H_2_:CO_2_ time course experiments over a period of 15 days. Compared to standard medium, omitting tungstate resulted in longer doubling time and lower final OD_545_, while multiplying tungstate concentration by 10 accelerated growth and increased final OD_545_ by ca. 1.2-fold (Fig. 2a; Table 1). Ethanol production was increased when tungstate concentration was augmented from 0 to 1× to 5× to 10×. Higher tungstate augmentation to 50× and 100× did not have further impact on ethanol production (Table 1).

**Table 1:** Growth parameters, acetate, and ethanol production of H_2_:CO_2_-grown *S. ovata* with different concentrations of tungstate

WO_4_^2−^	Growth parameters		Production rate (mmol gCD W^−1^ d^−1^)		Final concentration (mM)	
	Final OD_545_	Doubling time (h)	Acetate	Ethanol	Acetate	Ethanol
0×	0.32 ± 0.03	30.90 ± 0.60	4.16 ± 0.02	0.16 ± 0.11	39.94 ± 0.21	1.53 ± 1.03
1×	0.37 ± 0.03	29.07 ± 0.53	3.61 ± 0.14	0.41 ± 0.11	37.52 ± 1.49	4.29 ± 1.18
5×	0.40 ± 0.01	28.77 ± 0.92	3.06 ± 0.18	0.71 ± 0.06	34.59 ± 2.40	9.74 ± 0.79
10×	0.44 ± 0.02	28.52 ± 0.53	3.20 ± 0.22	1.21 ± 0.10	33.02 ± 1.98	13.15 ± 1.18
50×	0.45 ± 0.02	28.03 ± 0.35	3.16 ± 0.19	0.94 ± 0.34	34.09 ± 2.10	10.17 ± 3.63
100×	0.45 ± 0.04	28.30 ± 0.57	3.55 ± 0.23	1.01 ± 0.08	34.08 ± 1.32	9.51 ± 0.44

Each value is the mean and standard deviation of three replicates

As indicated by Fig. 2b, acetate production by *S. ovata* is growth-dependent. In contrast, ethanol production started at the end of the exponential phase and continued through the stationary phase (Fig. 2b). A pH drop from initial 7.3 to 4.8 was also observed coinciding with the production shift from acetate to ethanol (Fig. 2a). This production shift by *S. ovata* as well as the pH change in the medium followed the same trend described for other bacterial species including acetogens [10, 27]. In those bacteria, fatty acids (e.g., acetate) are produced first during an acidogenic phase, and solvents (e.g., ethanol) are generated in a second phase termed solventogenic phase when cell growth decreases and the pH become more acid [28].

Furthermore, the important increase in ethanol especially in the presence of 10× tungstate was accompanied with a notable decrease of acetate (Figs. 1a and 2b). This suggests the production of ethanol by *S. ovata* via acetate re-assimilation. For instance, the model solvent-producing bacterium *Clostridum acetobutylicum* can produce the solvents acetone, ethanol, and 1-butanol by re-assimilating fatty acids acetate and butyrate generated in the acidogenic phase, which resulted in an observable concentrations decrease in these fatty acids [28, 29]. A similar phenomenon was also observed for the acetogens *C. ragsdalei* and *C. ljungdahlii* during syngas fermentation where ethanol production probably occurs via acetate reduction [30].

### Acetate and ethanol production by MES with optimized medium

**Fig. 3:**
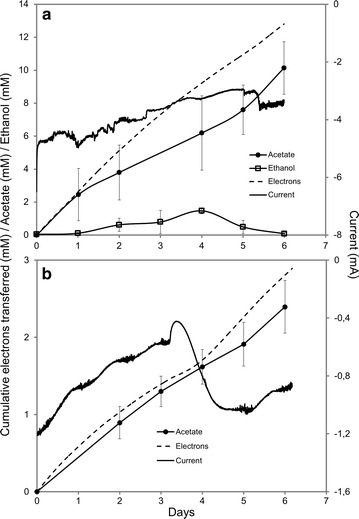
*S. ovata-*driven microbial electrosynthesis. Electron transferred, acetate, ethanol, and current production with **a** 10× tungstate and **b** 1× tungstate. Current data shown are from a representative example of three replicate MES reactors. Each curve for acetate and ethanol production is the mean of three replicates. No ethanol was detected with 1× tungstate

One of the most promising applications of *S. ovata* as a microbial catalyst is the reduction of CO_2_ into organic carbon products with electrons derived from the cathode of a MES system [4]. Thus, the impact of increasing tungstate concentration on acetate and ethanol production by *S. ovata*-driven MES was investigated (Fig. 3). A steady consumption of current over time with the concomitant production of acetate and ethanol was observed with 10× tungstate (Fig. 3a). The MES production of acetate was increased 4.4-fold from 32.0 ± 1.7 to 141.2 ± 56.6 mmol m^−2^ day^−1^ (380.0 ± 20.0–1694.5 ± 678.6 μM day^−1^) with 10× tungstate compared to 1× tungstate. These results show that increasing tungstate concentration improves acetate production from CO_2_ by MES, which may be caused by higher enzymatic capacity in the WL pathway for reactions like the conversion of CO_2_ to formate by tungsten-containing FDH. As expected, no acetate or ethanol was produced with a disconnected MES reactor control with 10× tungstate over a period of 15 days confirming that the metabolism of *S. ovata* was driven by current consumption.

1.5 ± 0.5 mM (*n* = 3) of ethanol was produced during the MES process when tungstate concentration was decupled with a production rate of 4.0 ± 1.2 mmol m^−2^ day^−1^ (48.0 ± 14.6 μM day^−1^) from day 1 to 4 (Fig. 3a). When unmodified 311 medium was used as the electrolyte, no ethanol production by MES was detected (Fig. 3b). To the best of our knowledge, production of ethanol by a pure culture-driven MES system has never been reported until now. Electron recovery in both acetate and ethanol with 10× tungstate was 87.6  ±  6.5 %. However, ethanol did not continue to accumulate over time in the MES system and started to decrease after day 4 (Fig. 3a). There are two possibilities to explain the concentration fluctuation observed with ethanol in *S. ovata*-driven MES system. First, ethanol could be abiotically lost mainly via evaporation from the MES system. Ethanol is a solvent and the MES reactor used in this study is an open system with constant N_2_:CO_2_ gas flushing in the liquid phase. Second, ethanol was biologically reoxidized by *S. ovata*. To evaluate abiotic loss of ethanol from the MES reactor, a control experiment was set up and 10 mM of ethanol was added to sterile 311 medium in the cathodic chamber with continuous input of N_2_:CO_2_ gas mixture over 7 days. Under these conditions, ca. 40 % of ethanol was lost. This observation suggests that the real ethanol production rate from day 1 to 4 in our MES system is underestimated. It may also explain in part why ethanol disappeared after day 4. These results do not exclude the second possibility that *S. ovata* could reoxidize the produced ethanol. This would be in accordance with the fact that *S. ovata* can grow with ethanol as sole substrate and that its genome harbors 5 genes coding for ADHs that could catalyze the conversion of ethanol to acetaldehyde using NAD^+^ or NADP^+^ as a cofactor [31, 32].

Ethanol production by the wild-type strain *S. ovata* DSM-2662 used here was recently reported for the first time during gas fermentation in a gas liquid (GLC) contactor system with a continuous flow of H_2_ and CO_2_. High yield of acetate was also obtained with the GLC system. In Blanchet et al. [33] study, yeast extract was added to the 311 standard cultivation medium, which probably resulted in bacterial cultures with higher cell densities contributing to the high productivity observed. However, utilization of yeast extract in the GLC system makes performance comparison difficult, since no yeast extract was added to other *S. ovata*-driven bioproduction processes from CO_2_ including MES [3, 25, 26, 34–36]. More surprisingly, Blanchet et al. indicated that in their study yeast extract was essential for the growth of *S. ovata* DSM-2662, although autotrophic growth without yeast extract is a well-established defining characteristic of this strain [3, 37].

### Involvement of AORs in the conversion of acetate to ethanol in *S. ovata*

**Fig. 4:**
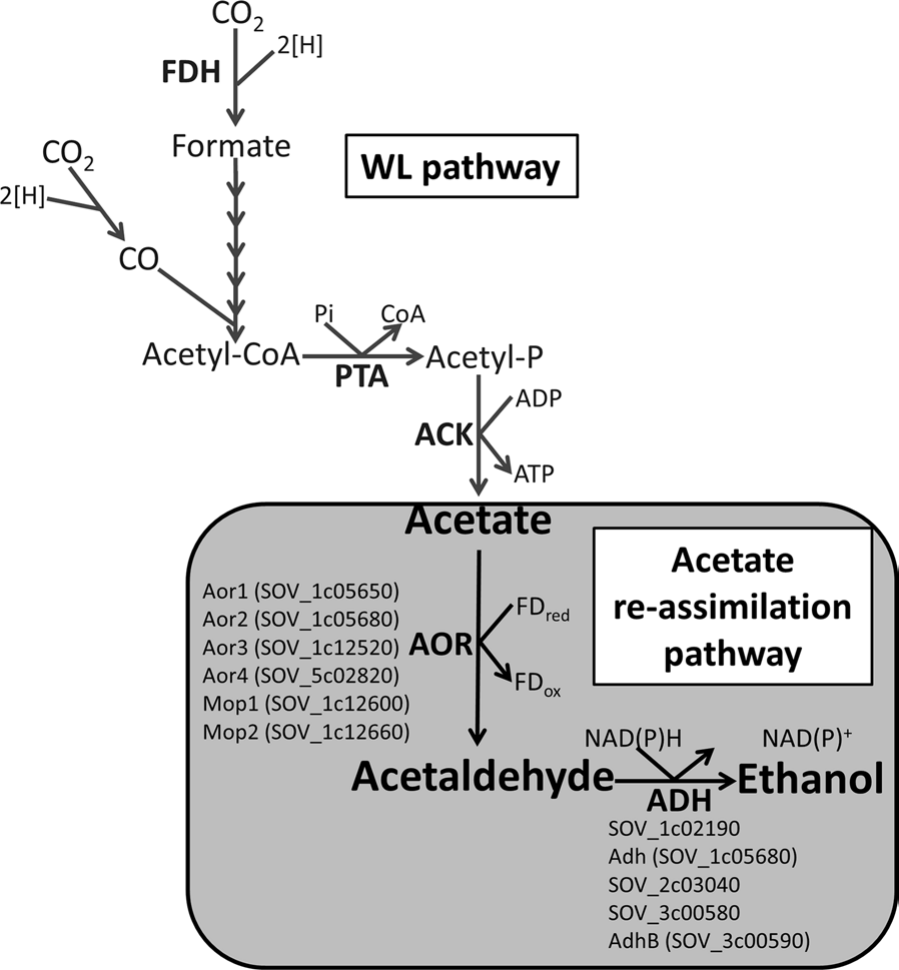
Ethanol biosynthesis via energetically favorable acetate re-assimilation in *S. ovata*. 6 putative AORs could be involved in the conversion of acetate to acetaldehyde and 5 putative ADHs could catalyze the production of ethanol from acetaldehyde. *Fd*_*red*_ reduced ferredoxin; *Fd*_*ox*_ oxidized ferredoxin

Production of ethanol by acetogens from H_2_ and CO_2_ has been predicted to have a positive energy balance, if acetate produced from acetyl-CoA is re-assimilated by an AOR to acetaldehyde that will be in turn reduced to ethanol by an ADH (Fig. 4). There is another well-known pathway for ethanol synthesis from acetyl-CoA usually found in heterotrophic microorganisms, which is mediated by a bifunctional aldehyde/alcohol dehydrogenase. However, this pathway would not be energetically favorable in acetogens growing with H_2_:CO_2_ [21]. Thus, for the autotrophic production of ethanol by *S. ovata*, AOR is likely involved. This is supported by the observation presented in this study that optimal ethanol production is dependent on the concentration of tungstate in the medium, since AORs use tungsten as a cofactor [38].

**Fig. 5:**
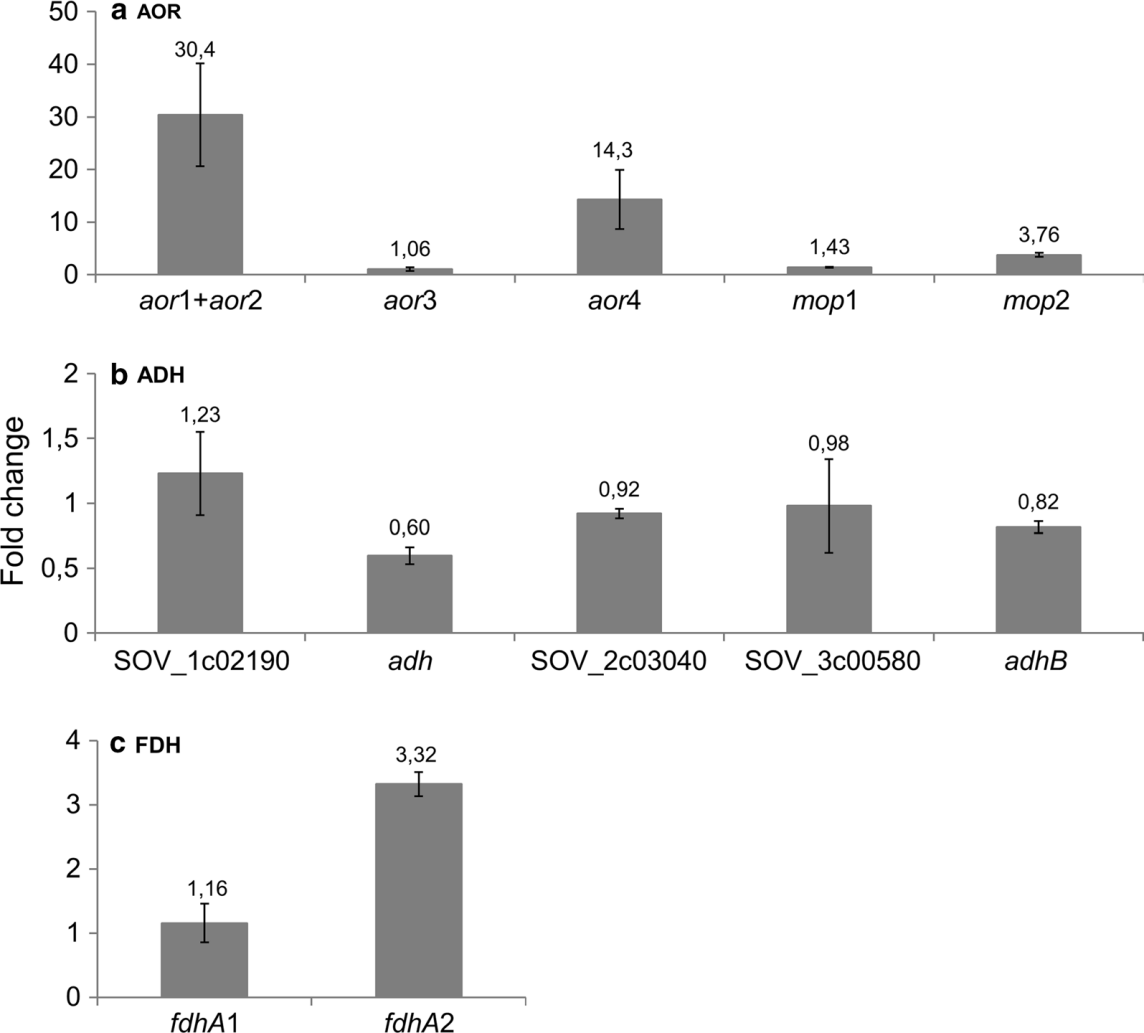
Transcript abundance fold change of **a** AOR-coding genes, **b** ADH-coding genes and **c** FDH α-subunit-coding genes in the presence of 10× tungstate versus 1× tungstate. *aor*1, *aor*2, *aor*3, *aor*4, *mop*1, and *mop*2 are coding for AORs. SOV_1c02190, SOV_2c03040, and SOV_3c00580 are annotated as class IV ADH. *adh* is annotated as an iron-type ADH and *adhB* is annotated as ADH2. *fdhA*1 and *fdhA*2 are coding, respectively, for α-subunits of FDH1 and FDH2. Results shown are from at least three replicates for each condition

Six genes annotated as AOR are found in *S. ovata* genome: *aor1* (SOV_1c05650), *aor2* (SOV_1c05680), *aor3* (SOV_1c12520), *aor4* (SOV_5c02820), *mop1* (SOV_1c12600), and *mop2* (SOV_1c12660) (Fig. 4) [32]. All six AORs are predicted to be tungsten-containing enzymes. *S. ovata* also possesses five genes coding for ADH. To further understand the impact of tungstate concentration on the ethanol biosynthesis pathway in *S. ovata*, RT-qPCR was conducted to measure the transcript abundance of AOR- and ADH-coding genes in the exponential growth phase of *S. ovata* cultures growing on H_2_:CO_2_ (Fig. 5).

The sequence alignment of Aor1 and Aor2 showed that Aor2 (484 AA) is identical to the C-terminal part of Aor1 (from position 96 to 579 out of 579 AA). Attempts to develop a RT-qPCR assay targeting the 5′ gene region unique to *aor1* failed. Thus, transcript abundance variation was investigated using a pair of primers targeting both *aor*1 and *aor*2 (Additional file 1: Table S1). In the presence of 10× tungstate, transcript abundance of *aor*1–2 was significantly increased by 30.4 ± 9.8-fold. Moreover, *aor*4 and *mop*2 were also upregulated by 14.3 ± 5.6-fold and 3.8 ± 0.4-fold, respectively (Fig. 5a). In contrast, no significant change in the expression of *aor*3 and *mop*1 was observed. Transcript abundance of the five genes coding for ADHs also remained the same in the presence of 1× or 10× tungstate (Fig. 5b). Results presented here suggest that tungstate was in limiting concentration in standard 311 medium and optimizing tungstate concentration enabled higher expression of the AORs encoded by *aor1*, *aor2*, *aor4,* and *mop2*, which subsequently augmented the enzymatic capacity of *S. ovata* for the conversion of acetate to acetaldehyde possibly leading to higher ethanol production.

### Transcript abundance of genes coding for tungsten-containing FDH

Among the metalloenzymes involved in the WL pathway, tungsten is known to be a cofactor for acetogenic FDHs [16, 39, 40]. Hence, RT-qPCR was employed to determine the impact of augmenting tungstate concentration under autotrophic conditions on the transcript abundance of genes *fdhA*1 (SOV_1c07830) and *fdhA*2 (SOV_3c08790). *fdhA*1 and *fdhA*2 are coding for α-subunits of the two FDHs of *S. ovata.* Transcript abundance of *fdhA*2 was increased 3.3 ± 0.2-fold when tungstate concentration was decupled, whereas the expression of *fdhA*1 remained the same (Fig. 5c). Augmentation of the expression of FdhA2 in *S. ovata* may cause increase in the carbon flux from CO_2_ to acetyl-CoA via the WL pathway and thus contributes to the improved growth as well as the higher ethanol production associated with optimal tungstate concentration.

### The conversion of longer carbon chain fatty acids to alcohols by *S. ovata* during gas fermentation

AORs can convert multiple fatty acids other than acetate including propionic and butyric acids to their respective aldehydes, which will be subsequently converted to alcohols [30]. The presence of six AORs in *S. ovata* suggests that this acetogen has the enzymatic potential for the biosynthesis of longer alcohols than ethanol. To determine if other fatty acids could be converted to aldehydes and then alcohols by *S. ovata* and also to verify if these reactions are associated with the presence of tungstate like the production of ethanol, 5 mM of propionic acid or 5 mM of butyric acid was added to *S. ovata* cultures under H_2_:CO_2_ growth condition in the presence of 1× or 10× tungstate concentration. Addition of propionate resulted in the generation of 1-propanol, whereas addition of butyrate led to the synthesis of 1-butanol (Table 2). Conversion of propionate to 1-propanol reached a final efficiency of 20.6 ± 4.1 % in a standard tungstate concentration medium after 380 h. When 10× more tungstate was present in the medium, conversion efficiency was increased by 1.6-fold (Table 2). Similarly, a higher conversion of butyric acid to 1-butanol was observed with decupled tungstate and the conversion efficiency was increased by 1.3-fold compared with standard tungstate medium (Table 2). Unpaired *t* test was used to evaluate the statistical significance of the fold increases in conversion efficiency observed in the presence of 10× tungstate. The *p* values for both fold changes in propanol and 1-butanol production was below 0.05 confirming statistical significance. These results indicate that *S. ovata* possesses AORs and ADHs that can convert multiple fatty acids to alcohols in a tungsten-associated manner. It also shows that *S. ovata* has the metabolic capacity for the reduction of longer fatty acids than acetate into alcohols, which could be harnessed to develop novel bioproduction processes.

**Table 2:** Conversion of propionate and butyrate to alcohols by H_2_:CO_2_-grown *S. ovata* with different concentrations of tungstate

WO_4_^2−^	Final concentration (mM)		Conversion efficiency (%)	
	1-propanol	1-butanol	Propionate to 1-propanol	Butyrate to 1-butanol
1×	1.03 ± 0.20	0.81 ± 0.13	20.64 ± 4.14	16.32 ± 2.79
10×	1.65 ± 0.14	1.08 ± 0.06	33.05 ± 2.91	21.59 ± 1.11

Each value is the mean and standard deviation of three replicates

## Conclusion

Increasing interest for acetogens in the recent years is mainly driven by the development of new technologies aiming to use CO_2_ waste gases as feedstock for the production of valuable organic carbon compounds [4, 41]. In this study, a simple optimization of tungstate concentration in *S. ovata* cultivation medium significantly improved the conversion of CO_2_ to acetate by MES and also promoted the production of ethanol during autotrophic growth on H_2_:CO_2_ or by MES. Furthermore, increasing tungstate concentration resulted in higher transcript abundance of genes coding for AORs strongly suggesting their participation in the ethanol biosynthesis pathway of *S. ovata*. In addition, a gene coding for tungsten-containing FDH was upregulated, indicating that augmenting tungstate concentration in the medium possibly increased the carbon flux in the WL pathway of *S. ovata* leading to increased growth and improved productivity. Successful conversion of longer carbon chain fatty acids to alcohols by *S. ovata* during gas fermentation reveals that AORs and ADHs of *S. ovata* can participate to the biosynthesis of longer alcohols than ethanol, and it also highlights the potential of this species for the bioproduction from CO_2_ of valuable chemicals other than acetate and ethanol. Further understanding and optimization of the AOR-associated pathways of *S. ovata* involved in alcohols production from fatty acids could lead to the bioengineering of highly efficient biocatalysts for the production of biofuels via MES or via other types of biosystems.

## Methods

### Bacterial strain, growth conditions, media modifications, and fatty acids conversion

The bacterium *S. ovata* DSM-2662 [31] was obtained from the Deutsche Sammlung Mikroorganismen und Zellkulturen (DMSZ). *S. ovata* cultures were routinely grown under a H_2_:CO_2_ (80:20) atmosphere (1.7 atm) at 30 °C in stoppered and crimp-sealed tubes. The medium DSMZ 311 used for cultivation was prepared under strict anaerobic condition. Yeast extract, casitone, betaine, resazurin, and sodium sulfide were all omitted from 311 medium. Trace elements investigated in this study are presented in Table 3 with their standard 1× concentrations in 311 medium. Growth was determined by measuring optical density (OD) at 545 nm with an Evolution™ 202 UV–Visible spectrophotometer (Thermo Scientific, Denmark). Conversion of fatty acids to alcohols by *S. ovata* during gas fermentation was carried out in 311 medium containing 5 mM propionic acid or 5 mM butyric acid and 0.01 µM or 0.1 µM of tungstate. All experiments were conducted at least in triplicate.

**Table 3:** Trace element concentrations (1×) in the standard 311 medium

Trace elements	Concentration (µM)
Na_2_MoO_4_·2H_2_O	0.15
NiCl_2_·6H_2_O	0.10
ZnCl_2_	0.51
FeCl_2_·4H_2_O	7.54
Na_2_SeO_3_·5 H_2_O	0.01
Na_2_WO_4_·2H_2_O	0.01

### Electrosynthesis of acetate and ethanol by MES

MES experiment was operated at 25 °C in a dual-chambered H-type reactor consisting of three-electrode with *S. ovata* inoculated in the cathodic chamber as previously described [3, 25]. The cathodic chamber and the anodic chamber were filled with a final volume of 300 ml of 311 medium containing 1× or 10× tungstate. The two chambers were separated by a Nafion 115 membrane (Ion Power, Inc., New Castle, DE, USA). Both the anode (36 cm^2^), and the cathode (36 cm^2^) were graphite sticks suspended in the culture medium. 100 ml of *S. ovata* cells pre-grown in 311 medium with 1× or 10× tungstate under a H_2_:CO_2_ atmosphere was injected into the cathodic chamber containing 200 ml of 311 medium with corresponding tungstate concentration. The cathode potential was set at −690 mV versus SHE with a potentiostat (ECM8, Gamry Instruments, PA, USA). During MES, both chambers of H-type reactors were continually bubbled with N_2_:CO_2_ (80:20). MES experiments were repeated in triplicate. Acetate and ethanol production rates were normalized with respect to the projected surface area of the graphite stick cathode.

### Analytical methods

Acetate, propionate, and butyrate were measured with an Ultimate 3000 high-pressure liquid chromatography system (Thermo Scientific, Denmark) equipped with an Aminex HPX-87H anion exchange column (Bio-Rad, California) set at a temperature of 30 °C. 5 mM H_2_SO_4_ was used as the mobile phase at a flow rate of 0.6 ml/min. Ethanol, 1-propanol, and 1-butanol were quantified with a Trace 1300 gas chromatograph (Thermo Scientific, Denmark) equipped with a flame ionization detector with the temperature set at 300 °C. Samples were injected into an Rtx-Wax column (fused silica, 30 m × 0.25 mm × 0.25 µM). (Restek, PA, USA). The injector port temperature was set at 250 °C. The oven temperature was programmed to 40 °C for 2 min, followed by an increase of 10 °C per minute until reaching 200 °C. All results presented in this study are from at least three independent experiments.

### Quantitative reverse transcription PCR (RT-qPCR)

Transcript abundance of AOR-, ADH-, and FDH α-subunit-coding genes was compared by RT-qPCR in *S. ovata* cultures grown under a H_2_:CO_2_ atmosphere in 311 medium containing 1× or 10× tungstate. AOR-coding genes in *S. ovata* DSM-2662 are *aor1* (SOV_1c05650), *aor2* (SOV_1c05680), *aor3* (SOV_1c12520), *aor4* (SOV_5c02820), *mop1* (SOV_1c12600) and *mop2* (SOV_1c12660). ADH-coding genes are SOV_1c02190, *adh* (SOV_1c05680), SOV_2c03040, SOV_3c00580 and adhB (SOV_3c00590). FDH α-subunit-coding genes are *fdhA1* (SOV_1c07830) and *fdhA2* (SOV_3c08790). Expression of these genes were normalized with *polC*1 (SOV_1c12130), a housekeeping gene constitutively expressed under the tested conditions coding for the DNA polymerase III. Primer 3 software was used to design primers for RT-qPCR (Additional file 1: Table S1) [42]. Total RNA was extracted from triplicate *S. ovata* cultures in the exponential growth phase after 3 days. Briefly, bacterial cultures were centrifuged and cell pellets were suspended in Trizol^®^Max Bacterial Enhancement Reagent (Ambion). Extraction of Total RNA was done with Trizol^®^ Reagent (Ambion) according to manufacturer’s instructions. The absence of DNA contamination in total RNA extracts was verif1ied by the absence of PCR amplification with the polIII-F and polIII-R primers. cDNA was generated with Superscript III Reverse Transcriptase (Invitrogen) using random primers. RT-qPCR was carried out with Brilliant III Ultra-Fast SYBR^®^ Green qPCR Master Mix (Agilent Technologies). Each RT-qPCR reaction was carried out at least in triplicate with an Mx3005P qPCR system (Agilent technologies). Relative expression levels of different genes were measured using Pfaffl mathematical model for relative quantification [43]. Fold changes over 2 or under 0.5 were considered significant transcript abundance variations.

## Additional file

10.1186/s13068-016-0576-0 Primers for RT-qPCR.

## Abbreviations

ADH: alcohol dehydrogenase
AOR: aldehyde ferredoxin oxidoreductase
BES: bioelectrochemical system
CODH/ACS: dehydrogenase/acetyl-CoA synthetase
FDH: formate dehydrogenase
GLC: gas–liquid contactor
MES: microbial electronsynthesis
OD: optical density
RT-qPCR: quantitative reverse transcription PCR
WL: Wood–Ljungdahl pathway
