# Supplementary material for: Effect of tungstate on acetate and ethanol production by the electrosynthetic bacterium Sporomusa ovata
Source: Biotechnol Biofuels. 2016 Aug 4;9:163. doi: 10.1186/s13068-016-0576-0 (PMC4973070; doi:10.1186/s13068-016-0576-0)
Supplement: Supplementary file 1 — 10.1186/s13068-016-0576-0 Primers for RT-qPCR. [file 13068_2016_576_MOESM1_ESM.docx]

**Supplementary table**

**Biofuels production by the electrosynthetic bacterium *Sporomusa ovata***

Fariza Ammam**^1^**, Pier-Luc Tremblay**^1^***, Dawid M. Lizak**^1^** and Tian Zhang**^1,2^***

**^1^***The Novo Nordisk Foundation Center for Biosustainability, Technical University of Denmark, DK-2970 Hørsholm, Denmark*

**^2^***School of Chemistry, Chemical Engineering and Life Science, Wuhan University of Technology, Wuhan 430070, PR China*

* Corresponding authors

| **Table S1.** Primers for RT-qPCR. | |
| --- | --- |
| Primers | Sequences 5´→3´ |
| adh_F | AGC TGA TTA CAT TAA ACC TAT GGG A |
| adh_R | TAG AAG CAA CAA GAG CAA TAC CTT T |
| adhB_F | TAC GAA GGC TAT AAC AAG ATT ACC C |
| adhB_F | TTC TAC TGA ATG GGA AAC AAC ATC T |
| SOV_1c02190_F | ATTGATAATGTTCCTGGTATGATTGTT |
| SOV_1c02190_R | AATTTGTTACGATCTTCTTCTGTCATT |
| SOV_2c03040_F | CCATTTTGGTATGTTTTGATTTTATTT |
| SOV_2c03040_R | AAAGGAAGAGAGCTTATAGTAGTTCCA |
| SOV_3c00580_F | AAGTGGATTAAAAATGATGGGCATAG |
| SOV_3c00580_R | ATGTACCTTTTCTTGGATTGGTTAAAG |
| fdh1_F | CAT CCG TGT AGA CAA GTC AGC TAT AA |
| fdh1_R | TGA TAA CCG GAA TAA ACA TTA GGT AAA |
| fdh2_F | TGA TAA CCG GAA TAA ACA TTA GGT AAA |
| fdh2_R | TGG TAG AGA AAT ATA CCC CTG AAT ATG |
| mop1_F | GGC GTA ATG TTA TAG ATG ATT TCC AT |
| mop1_R | CAT GAA GAC AGG TAA GGC TAA AGT AGT |
| mop2_F | ATA TTA CTA CCA TTG AAG GAT TGG CTA |
| mop2_R | ATA ACA GCA TCG ACT AAA GGT TTG |
| aor1+aor2_F | F TTG CTA CAA ACA TAA AAG TGG |
| aor1+aor2_R | CGA AGG CAA TAA AGA GAC ACA TAC |
| aor3_F | ACA AGT GAG GTT CCA GAA AAG TAT G |
| aor3_R | CGG ACT CTT ACC ACC TAC AGA GAG |
| aor4_F | GGA CAG TTC GAT AAT ACC CTC TTT T |
| aor4_R | GTT TGA CCT ACG CTA CCA CTC C |
| polIII_F | TCAACTAAAGAGAAGAGTATCCAAACGA |
| polIII_R | GAGGTAAAAGCCCATTATCAACTATCA |
